# Long-term patient satisfaction and removal rate after essure sterilization: a follow-up study

**DOI:** 10.1186/s12905-022-01838-3

**Published:** 2022-06-23

**Authors:** Branka Žegura Andrić, Maja Rosič, Tamara Serdinšek, Rok Šumak

**Affiliations:** 1 Department of General Gynaecology and Urogynaecology, Clinic for Gynaecology and Perinatology, University Medical Centre Maribor, Ljubljanska 5, 2000 Maribor, Slovenia; 2 Health Institution Rosič, Ptuj, Slovenia

**Keywords:** Essure, Hysteroscopic sterilization, Outpatient hysteroscopy, Satisfaction rate

## Abstract

**Background:**

The objective of our study was to assess the rate and causes for Essure® micro-insert system removal and patients’ long term satisfaction rate with the procedure.

**Methods:**

All patients who underwent Essure® hysteroscopic sterilization at our tertiary centre between years 2007 and 2018 were included in this follow-up study. A questionnaire was sent to all patients per standard mail. Patients who did not respond to questionnaires per mail, were called by phone. The satisfaction with the Essure® sterilization, as well as any additional procedures after the insertion or insertion-related complications were analysed.

**Results:**

From the year 2007 to 2018, we performed 427 Essure® hysteroscopic sterilizations and of these, 329 patients responded to the questionnaire (response rate 77%). Ten patients (3%) had Essure® removal, two of them due to pain (0.6%). Patients were very satisfied with the procedure (9.5 on scale 0–10). Most patients (95.3%) would recommend the procedure to their friend.

**Conclusions:**

Essure® hysteroscopic sterilization is a procedure with a very high satisfaction rate and a very low removal rate due to sterilization-related complications.

*Trial registration* Institutional review board of University medical centre Maribor approved the study, approval number UKC-MB-KME-73/19.

## Background

Hysteroscopic sterilization using the Essure® (Conceptus, San Carlos, CA, USA) micro-insert system represents a permanent form of a female contraception. It was first introduced in Europe in 2001 and in the United States of America (USA) in 2002. [1] During the procedure, the Essure® micro-insert is hysteroscopically inserted into the proximal part of both fallopian tubes. [2] After the insertion, the polyethylene terephthalate fibres of the insert cause a local inflammatory reaction and fibrous tissue growth, which in turn surrounds the micro-insert in about 12 weeks, causes its anchoring to the lumen of the tube and subsequently obliteration of the tube. [3] In years 2002–2016, United States Food and Drug Administration (FDA) received more than 8000 Essure® sterilization-related complication reports*.* [4] Reported complications included gynaecological issues such as chronic pelvic pain and abnormal uterine bleedings as well as general health problems such as headache, fatigue, allergic reactions, and autoimmune diseases. As a response, FDA issued a request for additional post-marketing surveillance regarding the efficacy and safety of the Essure® system in 2016 and emphasized that patients should be counselled regarding the possible risks of the procedure. [5] Due to marketing reasons of the manufacturer, Essure® sterilization was withdrawn from the market in 2018. [6] However, there is still ongoing research regarding the safety and efficacy of the Essure® micro-insert system. The aim of our study was to determine the rate of Essure® micro-insert system removal, causes for micro-insert removal, and patients’ long-term satisfaction with the procedure.

## Methods

This was a questionnaire-based follow-up study in which we included all patients who underwent Essure® hysteroscopic sterilization at our Department of General Gynaecology and Urogynaecology, Clinic for Gynaecology and Perinatology, University Medical Centre Maribor between years 2007 and 2018. Institutional review board of University medical centre Maribor approved the study, approval number UKC-MB-KME-73/19.

At our department, hysteroscopic sterilization using Essure® micro-insert system was performed in the outpatient clinic by an experienced hysteroscopic surgeon. According to our legislation, all patients had to obtain a sterilization approval from the first-degree committee for pregnancy termination and sterilization. First-degree committee consists of Obstetrics and gynecology specialist that approves or declines woman’s wish for sterilization according to our legislation. From the moment committee approves permanent sterilization, at least 6 months have to pass before sterilization is performed (Essure® or laparoscopic sterilization).

Before the procedure, patients received a premedication with a 100 mg ketoprofen suppository. The procedure was performed in concordance with the manufacturer’s instructions. After the procedure, patients were discharged and a reliable form of contraception was advised, until tubal occlusion was confirmed 12 weeks after the procedure using either 2D transvaginal ultrasonography or hysterosalpingography.

In the year 2019, a closed-type questionnaire and informed consent form were sent to all patients per mail. A closed-type questionnaire contained questions in which participants were provided with options to choose a response from. The questionnaire was developed by our team. Pain in the questionnaire was assessed by using Numerical Rating Scale (NRS) from 0 to 10. A resident doctor, who was not involved in performing Essure® procedures, neither in writing this article, called our patients who did not respond per mail and filled the questionnaire with them per phone. The questionnaire contained questions regarding basic demographic information, their recall on pain level during the procedure on a visual-analogue scale from 0 (no pain at all) to 10 (unbearable pain), the satisfaction with the procedure on a scale from 0 (not satisfied at all) to 10 (completely satisfied), any additional procedures after insertion and insertion-related complications.

The aim of our study was to determine the rate and causes for Essure® micro-insert system removal and patients’ long-term satisfaction with the procedure. Data were analysed using SPSS Statistics Programme. Descriptive statistics and a non-parametric Kruskal–Wallis test were used. Statistical significance was set at *p*-value < 0.05.

## Results

Between years 2007 and 2018, 427 women underwent hysteroscopic sterilization with the Essure® micro-insert system at our facility. Out of these, 206 responded to our questionnaire (48.2% response rate). Another 123 answered to the questionnaire per phone, altogether 329. The overall response rate was 77%. Ten patients (3%) had Essure® removal and are presented in the Table 1.

**Table 1:** Patients with removed Essure®

Age at sterilisatio*n* [years]	Body mass index [kg/m2]	Number of deliveries [*n*]	Pain during the insertion [scale 0–10]	Satisfaction with the procedure [scale 0–10]	Time from the insertion to Essure® removal [months]	Reason for Essure® removal
41	29.4	2	0	0	72	Pain in the lower abdomen
40	23.4	2	5	0	84	Pain in the lower abdomen
47	24.5	2	7	0	6	Abnormal position of the insert
40	28.4	3	7	0	12	Abnormal position of the insert
32	19.9	2	9	3	1	During other gynecological procedure
43	21.6	2	5	10	72	During other gynecological procedure
33	23.0	1	2	8	30	During other gynecological procedure
40	23.3	2	1	10	60	During other gynecological procedure
38	20.6	2	9	5	1	Unilateral Essure insertion
37	25.0	2	3	10	10	Unilateral Essure insertion

Two removals were due to pelvic pain (0.6%), two because the tubes were still patent on routine check-up 12 weeks after insertion (0.6%), two because a bilateral insertion was not possible (0.6%) and four were removed during gynaecological procedures that were unrelated to the Essure® (1.2%). Basic patients’ characteristics are presented in the Table 2.

**Table 2:** Basic patients’ characteristics

	Mean ± SD	Range
Age of sterilisation [years]	40 ± 3.3	32–47
Body mass index [kg/m2]	25.6 ± 4.7	17–39.5
Number of deliveries	2.0 ± 0.7	0–4
Number of vaginal deliveries	1.8 ± 0.9	0–4
Number of caesarean deliveries	0.2 ± 0.5	0–3

**Fig. 1:**
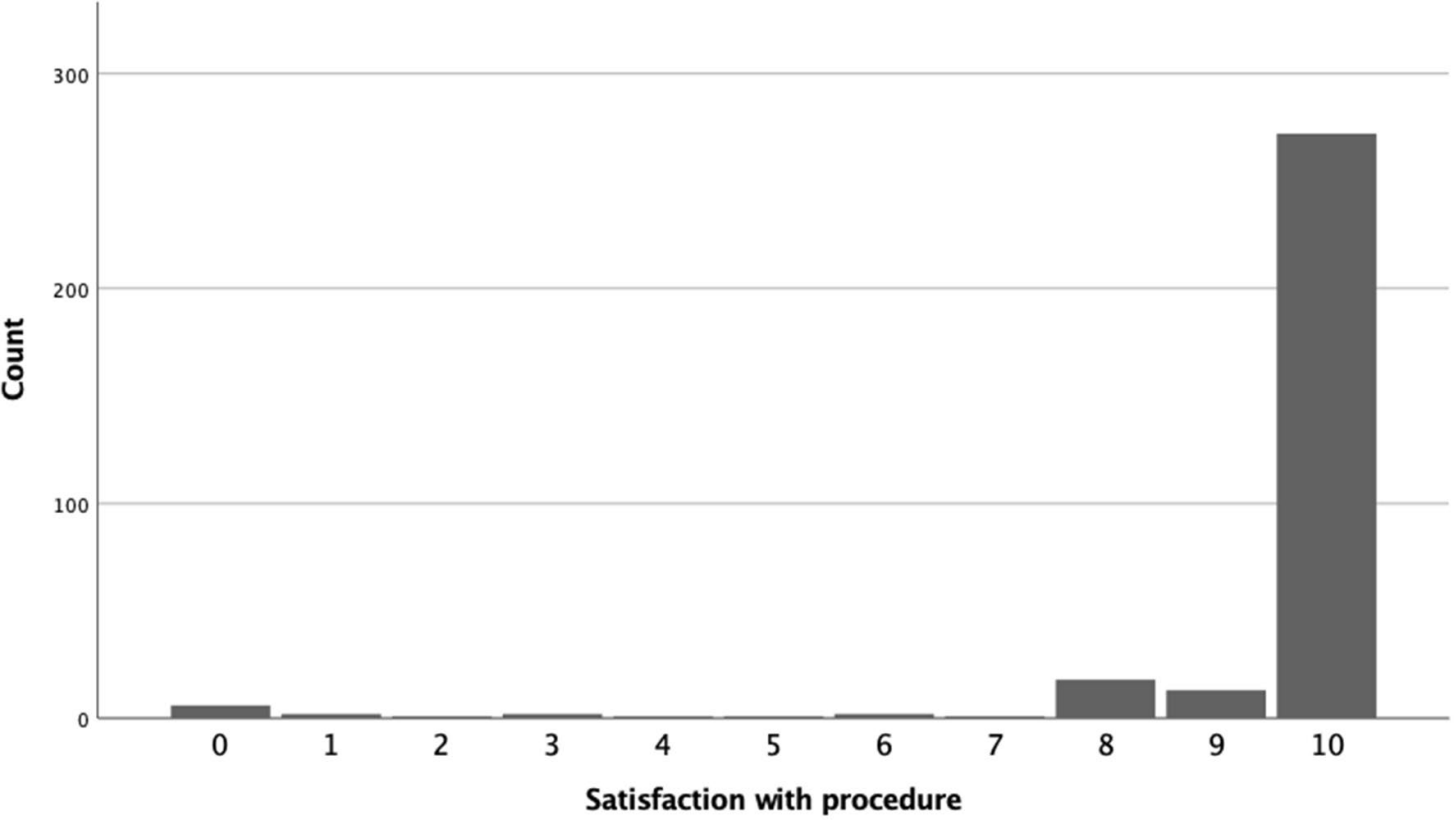
Patients' satisfaction with the procedure

The mean time from the Essure® procedure was 84.2 ± 26.6 months. The average pain level during the procedure using visual analogue scale was 3.65 ± 2.8 (range 0–10) and 73.9% of women evaluated the pain level as 5 or lower. The average satisfaction rate with the procedure was 9.5 ± 1.8 (range 0–10). As seen from the Fig. 1, 85.3% of patients evaluated their satisfaction rate as 10 (complete satisfaction) and 95% of patient evaluated their satisfaction rate as 8 or higher.

None of the following parameters were significantly associated with the satisfaction rate: body mass index (*p* = 0.720), number of vaginal deliveries (*p* = 0.664), age (*p* = 0.99). The pain level during the procedure (*p* = 0.009) was significantly associated with the satisfaction rate. Most patients (95.3%) would recommend Essure® sterilization procedure to their friends.

## Discussion

Sterilization is the most common contraceptive method among married couples and approximately twice as many couples choose female partner sterilization over male sterilization than vice versa [7]. Almost 30 years ago, World Health Organization (WHO) estimated that more than 100 million women worldwide rely on surgical sterilization for contraception [8]. Although female sterilization is more common, it is less effective, more costly and carries more risk when compared with male sterilization techniques. The primary concern guiding sterilization provision and policy should be the respect for an individual woman’s reproductive autonomy [7]. Besides that, the efficacy and safety of female sterilization techniques should be of uttermost importance when counselling women and performing the procedures themselves.

Since its introduction into clinical practice in 2001, several studies have confirmed the feasibility, safety and efficacy of the Essure® hysteroscopic sterilization technique. For example, in his review from 2005, Abbott estimated that the micro-insert can be delivered to more than 90% of tubes and has a 99% success rate of pregnancy prevention. Moreover, it can be performed in an outpatient setting and is acceptable to patients [1]. A study by Levy et al.estimated the 5-year cumulative pregnancy rate to be 2.6 per 1000 procedures, with most pregnancies occurring in women without appropriate follow-up. Misread hysterosalpingograms, undetected pregnancies before the procedure, and failure to follow product-labelling guidelines were some other causes for failure of the method [9]. Another two reviews from 2009 and 2010 also confirmed that the Essure® system appears to be a safe, permanent, non-invasive method of contraception with efficacy as high as 99.74% [3, 10].

The reported complications of Essure® sterilization in the initial literature were rare and most commonly included micro-insert malposition, chronic pain, unintended pregnancy, infection, and nickel allergy. Although rare, failure to diagnose and improperly placed micro-insert could be the cause of ineffective contraception or in some rare cases severe patient morbidity. However, as it was emphasized by Adelman et al., a common reason for failure to diagnose the Essure® malposition or pregnancy due to an properly placed device was physician and patient non-compliance with follow-up protocols [11]. In 14 years of its use in clinical practice, United States FDA received more than 8000 Essure® sterilization-related complication reports [4], from chronic pelvic pain, abnormal uterine bleedings and general health problems to allergic reactions and autoimmune diseases [5]. This was followed by an extensive media coverage on this topic and removal of the implant from the global market in 2016. Up to then, around 1 million Essure® units had been sold worldwide and the number of complication reports received by FDA rose to 15 000. Of these, six reports were relating to four adult deaths: first one reportedly due to post procedure group A Streptococcus infection, second due to uterine perforation during micro-insert placement, third due to an air embolism during the procedure and fourth from a suicide [6].

Although the Essure® micro-insert was removed from the market four years ago, there are still ongoing studies evaluating its long-term safety and efficacy. According to our results, Essure® hysteroscopic sterilisation is a procedure with a low removal rate. The satisfaction rate in our study was very high and almost all patients would recommend the procedure to their friend as well. This is in concordance with the results of some other studies that also confirmed that women are very much satisfied with Essure® sterilisation [12–16].

Regarding the complications and micro-insert-removal rate, we discovered that ten of our patients (3%) had Essure® removal and of those only two removals were pain related (0.6%). In comparison, in the post-market surveillance study, ordered by FDA, the removal rate of Essure® micro-insert was 6.8% [4]. Moreover, in the systematic review of Essure®-related complications from 2014, 100 cases of insert malposition, including perforation, expulsion or migration of micro-insert were reported. [11] We performed two Essure® removals because the tubes were still patent on routine check-up 12 weeks after the insertion (0.6%), two because a two-sided insertion was not possible (0.6%) and four were removed during gynaecological procedures that were unrelated to the Essure® (1.2%).

The association between the Essure® sterilization procedure and de novo chronic pelvic pain has also been the subject of several studies. While some studies discovered that it is very uncommon and as low as 0.16% [15], others found out that Essure® insertion is a risk factor for new onset of chronic pelvic pain [17, 18]. A retrospective cohort study performed on 458 patients showed acute pelvic pain in 8.1% and persistent pelvic pain 3 months after the procedure in 4.2% of patients. Procedures were performed under general anaesthesia, as well as in outpatient settings. Patients with history of any previous chronic pain such as chronic pelvic pain, lower back pain or fibromyalgia were more likely to experience both acute and chronic pain after micro-insert insertion [18]. Although there was no direct question about chronic pelvic pain in our questionnaire, two Essure® micro-inserts were removed due to chronic pelvic pain. Furthermore, all patients were invited to return to our clinic after the procedure in case any problems would occur. They also received contact information of the lead surgeon and none of them returned to our facility with a complaint of chronic pelvic pain. We believe that chronic pelvic pain as well as satisfaction with the procedure might depend on the surgeon’s experience and whether Essure® was inserted under general anaesthesia or in an outpatient setting. Our results show that the satisfaction with the procedure is related to the pain experienced during the procedure. It is of uttermost importance that Essure® insertion is performed in high frequency centre and by experienced surgeon. Multicentre study that included 1032 patients, showed that Essure® insertion is significantly less painful when performed by an experienced surgeon compared to an unexperienced one. [19]

The main limitation of our study is that it is questionnaire based and the second one is the relative long period between the procedure and the time the questionnaire was administered for assessment of pain during the procedure. Another limitation is, that no questions were asked about chronic pelvic pain, dysmenorrhea or changes in menstrual bleeding pattern. This study was focused only on removal rate, reasons for removal and satisfaction with the procedure. However, all patients were followed after the procedure and still have access to the surgeon who performed the procedure in case of any procedure related complications, at any time.

The main advantages of our study are a long follow-up rate and a standardized follow-up of all our patients. By performing a follow-up in all patients, we were able to confirm an obliteration of the tubes as well as the position of the micro-inserts, thus preventing any long-term complications due to the possible malposition of the micro-inserts. Moreover, all Essure® sterilization procedures at our department were performed by the same surgeon, experienced in outpatient hysteroscopy and complex hysteroscopic procedures. Just as Rosen et al.showed, hysteroscopic sterilization should be performed by experienced surgeons, as time of the procedure and insertion success rate improve with experience [20]. We believe that the promising results of our follow-up study could be greatly attributable to this fact.

## Conclusions

Our results demonstrate that the Essure® hysteroscopic sterilization is a procedure with a very high satisfaction rate. Most of the Essure® removals in our population were performed due to Essure® unrelated complaints. It is of uttermost importance that the procedure is performed by experienced surgeon and that the patients receive appropriate follow-up. We believe that the withdrawal of the Essure® system from the market might have occurred too soon. Only future studies with long term follow up and appropriate sample sizes will show whether this decision has deprived our patients of an efficient and safe sterilization method.

## Data Availability

The datasets used and/or analyzed during the current study are not publicly available, since questionnaires contain personal data of patients. Datasets are available from the corresponding author on reasonable request. Anonymized participant data used in the preparation of this article will be made available on request from the lead author.
